# Serum Cytokine Concentrations in a Patient with Rheumatoid Arthritis on Etanercept Therapy Who Subsequently Developed Pneumocystis Pneumonia: A Case Report

**DOI:** 10.1155/2011/185657

**Published:** 2011-12-15

**Authors:** Masao Sato, Masao Takemura, Ryuki Shinohe, Katsuji Shimizu

**Affiliations:** ^1^Department of Orthopaedic Surgery, Gifu University School of Medicine, 1-1 Yanagido Gifu, Gifu 501-1194, Japan; ^2^Department of Informative Clinical Medicine, Gifu University School of Medicine, 1-1 Yanagido Gifu, Gifu 501-1194, Japan; ^3^Department of Orthopadeic Surgery, Nishimino Welfare Hospital, 986 Oshikoshi Yoro, Gifu 503-1394, Japan

## Abstract

We report a rheumatoid arthritis patient who was treated with etanercept. Serum levels of tumor-necrosis-factor- (TNF-) *alpha*, soluble-tumor-necrosis-factor receptor- (sTNFR-) I and -II, interleukin- (IL-) 6, and IL-1 beta were measured by ELISA before and during the course of therapy. While the serum levels of IL-6 and IL-1 beta dropped rapidly following the initiation of therapy, the concentrations of TNF-alpha and sTNFR-II steadily increased to a plateau. Although significant clinical efficacy was observed, etanercept had to be discontinued when after 12 weeks of therapy the patient was found to have pneumocystis pneumonia.

## 1. Introduction

Rheumatoid arthritis (RA) is an autoimmune inflammatory disease in which cytokines, such as tumor-necrosis-factor- (TNF-) *alpha* and interleukin- (IL-) 1, are thought to play a major role in the pathogenesis. Consequently, several publications have focused on the serum and/or synovial fluid levels of various inflammatory cytokines in patients with RA, psoriatic arthritis, and ankylosing spondylitis, with the clinical utility of such measurements being advocated [1, 2]. Recently, TNF-*alpha* inhibitors have been found to induce a rapid and sustained attenuation of disease activity in patients with RA. We describe here a patient with RA treated with a TNF-*alpha* inhibitor, etanercept for 12 weeks before discontinuation of therapy for pneumocystis pneumonia (PCP). The serum concentrations of TNF-*alpha*, sTNFR-I and -II, IL-6, and IL-1 beta were measured before, during, and after etanercept therapy. 

## 2. Case Presentation

A 54-year-old Japanese man who had been diagnosed with RA for 8 years according to the 1987 American Rheumatism Association classification criteria for RA was admitted with polyarthritis. He complained of pain and swelling in the wrists, fingers, and knees and had difficulty walking without assistance. He claimed to have morning stiffness lasting about 3 hours. Past medical history included a left total knee arthroplasty complicated by a traumatic fracture of the left distal femur postoperatively and a cerebral infarction with residual right hemiparesis. His medication regimen included sulfasalazine (1000 mg/day), bucillamine (300 mg/day), mizoribine (150 mg/day), and prednisolone (10 mg/day). The Steinbrocker stage was IV and functional class was 4. He was unable to stand unassisted due to bilateral knee pain. Swelling and tenderness were present in the proximal interphalangeal and metacarpophalangeal joints of both hands and fingers, as well as in the wrists and knees. Right-sided muscle weakness was observed. Laboratory findings were as follows: white blood cell count 8800/mm^3^ (75.6% neutrophils, 19.5% lymphocytes, 2.9% monocytes, 0.3% eosinophils, 0.2% basophils), hemoglobin 10.7 mg/dL, platelets 48.7 × 10^4^/*μ*L, erythrocyte sedimentation rate (ESR) 75 mm/h, C-reactive protein (CRP) 3.58 mg/dL, rheumatoid factor (RF) 219.6 IU/mL, and anticyclic citrullinated peptide antibody (ACPA) 189 IU/mL. Serum *β*-D-glucan was negative. The purified protein derivative skin test for tuberculosis was negative. 

Treatment was initiated with subcutaneous injections of etanercept 25 mg twice a week. Blood samples were collected before the first dose (at baseline), 24 hours after the first injection (day 1), daily until day 8, and weekly thereafter. Specimens were centrifuged at 3000 rpm and the sera stored at −80°C until the assay was performed. TNF-*alpha*, TNFR-I and -II, IL-6, and IL-1 beta concentrations were measured using commercial ELISA kits.

After initiating etanercept, the patient's clinical condition improved markedly, with swelling and tenderness of the wrists and finger joints significantly better. He was able to stand with the aid of crutches despite residual hemiparesis. The duration of morning stiffness dropped to below 30 minutes. The 28-joint count Disease Activity Score (DAS28) dropped significantly within the first 4 weeks and continued to decrease (DAS28 = 8.09, 5.37, 4.73, and 4.60, at baseline, 4 weeks, 8 weeks, and 12 weeks, resp.), achieving the European League Against Rheumatism (EULAR) moderate response criteria. The CRP dropped to below 0.30 mg/mL within the first 2 weeks of therapy. Subsequently, the oral immunosuppressive regimen of sulfasalazine, bucillamine, and mizoribine was discontinued after initiation of treatment. Prednisolone was reduced to 5 mg/day.

**Figure 1:**
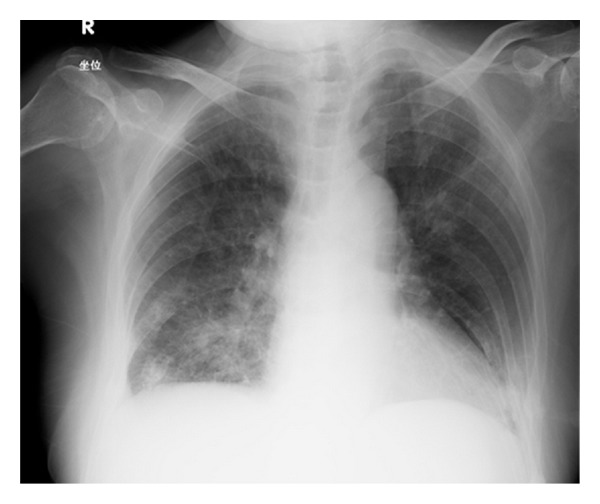
Chest radiograph revealed bilateral ground-glass infiltrates and reticular shadows.

After 12 weeks of treatment with etanercept, the patient developed a nonproductive cough along with a fever of 38°C. Serum *β*-D-glucan was found to be positive, and sputum polymerase chain reaction (PCR) was positive for pneumocystis (Figure 1). Etanercept was discontinued, and an antifungal regimen initiated.

**Figure 2:**
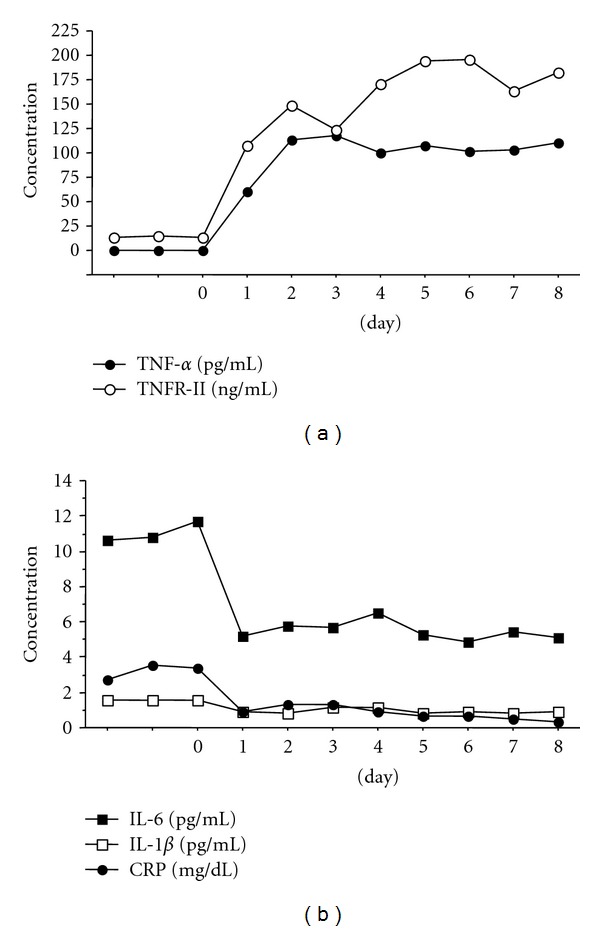
Time course of serum cytokines concentrations from baseline to 8 days. (a) TNF-*alpha* was not detectable in baseline samples. One day after the start of etanercept therapy TNF-*alpha* was measurable by ELISA. TNF-*alpha* levels became steady within 2 days of etanercept therapy. TNFR-II concentrations became elevated after the start of etanercept therapy. Within the first week of etanercept therapy, TNFR-II reached levels ranging from 160 ng/mL to 190 ng/mL. (b) Serum IL-6, IL-1 beta, and CRP concentrations were decreased just after the initiation of treatment.

**Figure 3:**
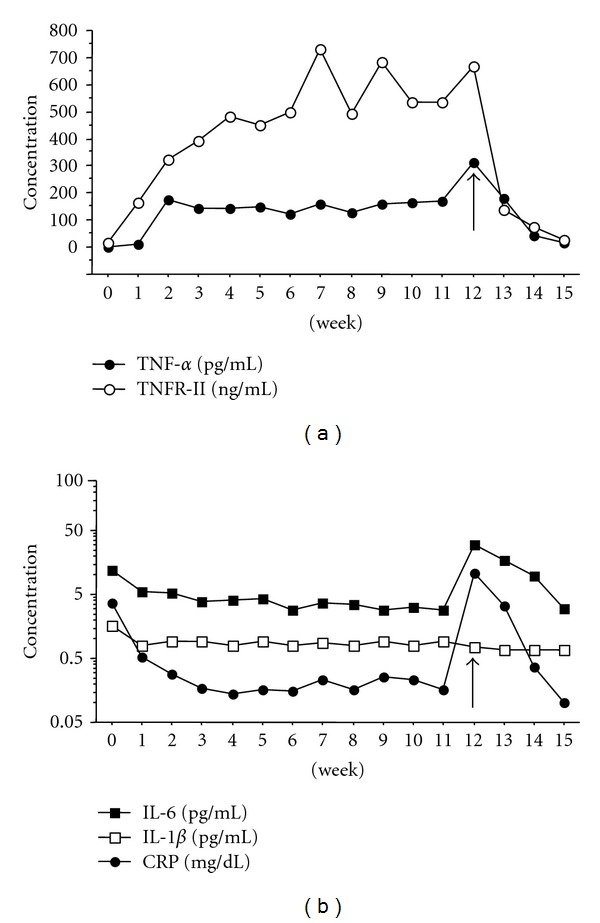
Time course of serum cytokines concentrations. (a) TNF-*alpha* concentration increased between weeks 1 and 2 after the initiation of etanercept therapy then remained steady ranging from 100 pg/mL to 170 pg/mL. At 12 weeks, the patient developed PCP and the TNF-*alpha* value increased steeply (arrow). With cessation of etanercept, the TNF-*alpha* concentration immediately returned to pretreatment levels. Four weeks after the initiation of therapy, the TNFR-II concentration became steady, ranging from 300 ng/mL to 700 ng/mL. With cessation of etanercept due to the PCP, TNFR-II concentration returned to pretreatment levels. (b) On the other hand, at week 12, the levels of IL-6, CRP, and TNF-*alpha* showed an acute peak (arrow), which tapered downward over the following 3 weeks with the institution of an appropriate antifungal regimen.

**Figure 4:**
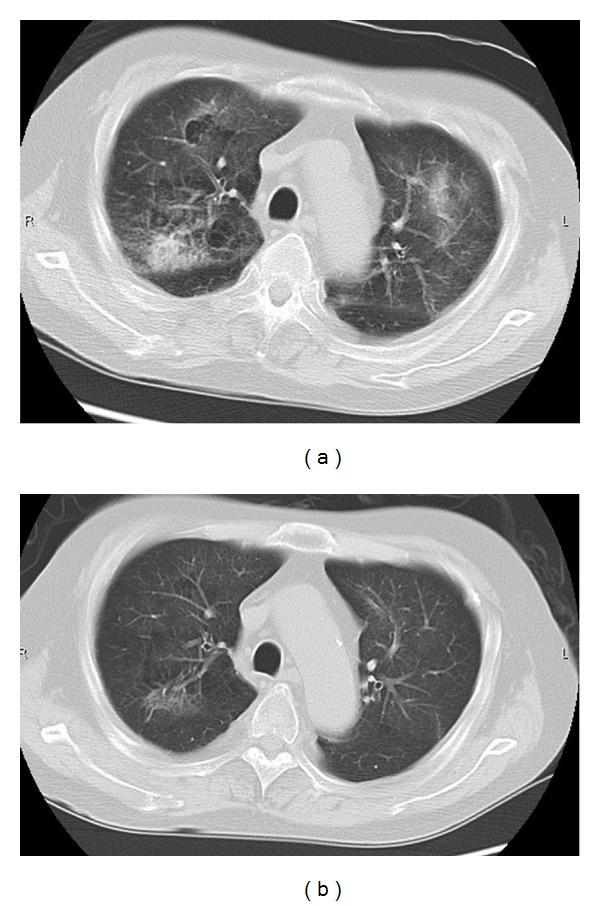
(a) Computed tomography scans on the day patient diagnosed as PCP showed infiltrates and reticular shadows. (b) The shadows disappeared after treatment with antifungal agents.

Preceding etanercept therapy, the serum IL-6 levels were found to be elevated, correlating with the high CRP value and high clinical disease activity. Conversely, the serum level of TNF-*alpha* was nondetectable (<0.2 pg/mL); the sTNFR-I, -II, and IL-1 beta values were not found to be significantly elevated (Figure 2). After initiation of etanercept, there was a rapid decline in the IL-6 and CRP levels, whereas the levels of TNF-*alpha* and sTNFR-II increased significantly within 24 hours after administration of the first dose to a plateau after 2 weeks (range: 100 pg/mL to 170 pg/mL) and 6-7 weeks (range: 300 ng/mL to 700 ng/mL), respectively. The sTNFR-I and IL-1-beta levels remained relatively unchanged (sTNFR-I levels at baseline, 3.56 ng/mL; 4 weeks, 3.36 ng/mL; 8 weeks, 2.93 ng/mL; 12 weeks, 3.58 ng/mL). At week 12, concurrent to the patient developing clinical symptoms of PCP, the levels of IL-6, CRP, and TNF-*alpha* showed an acute peak, which tapered downward over the following 3 weeks with the institution of an appropriate antifungal regimen (Figures 3 and 4).

## 3. Discussion

Several cytokines, chemokines, and matrix metalloproteinases with serum levels that correlate with the disease activity of RA have been reported. Furthermore, a favorable clinical response following the administration of TNF-*alpha* inhibitors was shown to be accompanied by the reduced serum levels of these inflammatory mediators [3].

Etanercept is a recombinant fusion protein consisting of two p75 receptors and the Fc domain of the human IgG1 that binds to both soluble and cell-bound TNF-*alpha* [4]. Although etanercept is known to reduce disease activity and inhibit bone and joint destruction in patients with RA [4], it is not clear if and how etanercept modulates the serum levels of TNF-*alpha*, TNFR-I, and TNFR-II. We previously demonstrated that serum TNFR-II concentrations in RA patients (*n* = 45) not on biologics were significantly higher than in healthy controls (*n* = 80) (7.28 ± 4.16 ng/mL versus 4.40 ± 0.88 ng/mL) [5]. In the patient reported here, the serum TNFR-II concentration was elevated to more than 500 ng/mL during treatment with etanercept, with return to baseline levels after discontinuation of treatment. Since etanercept is composed in part by the p75 TNF receptor, the ELISA assay might be detecting (a component of) etanercept as TNFR-II.

 TNF-*alpha* concentrations were elevated within 24 hours of etanercept therapy in the present patient and remained high until the cessation of etanercept treatment, which supports previous reports that TNF-*alpha* concentrations are elevated during etanercept therapy [6]. We could explain this rise in serum TNF-*alpha* by the possibility that the assay might be measuring TNF-*alpha* that is bound to etanercept. Etanercept has an approximately 50-fold greater affinity for human recombinant TNF-*alpha* in a binding inhibition assay and is approximately 1000 times more efficient than monomeric soluble TNFR. In addition, etanercept has a 5- to 8-fold longer plasma half-life compared with naturally occurring soluble TNFR. [7, 8]. Etanercept-bound TNF-*alpha* is known to be immunoreactive although without biological activity [6]. In this case, even though TNF-*alpha* as measured by the ELISA was elevated during the course of treatment, disease activity was well inhibited. The levels of CRP were clearly lower during the course of treatment.

 Mori et al. reported that etanercept lowered the TNFR-I, IL-6, and IL-1 beta serum concentrations in juvenile idiopathic arthritis patients. The concentrations of IL-6 at weeks 2, 4, 8, and 12 of IL-1 beta at week 4 and of TNFR-I at week 12 were significantly lower than before treatment [9]. Our data did not show remarkable changes in TNFR-I concentrations, but serum IL-6 and IL-1 beta concentrations were markedly decreased during the course of treatment. Trials have shown that etanercept therapy had significant clinical efficacy in patients with RA [10, 11]. It was suggested that clinical efficacy of etanercept was due to the reduction in CRP, IL-6, and IL-1 beta concentrations. 

 In general, therapies using biological agents have been highly effective in the treatment of RA. However, cases with serious adverse events have also been reported. Biologics may mask the clinical symptoms of infection because they diminish the production of acute inflammatory proteins [12]. In the case discussed here, CRP and IL-6 levels were found to be elevated when the patient developed PCP. Furthermore, the serum concentration of TNF-*alpha* increased by more than 300 pg/mL. Previously reported was a patient with high concentrations of TNF-*alpha* on etanercept therapy with development of gastric outlet obstruction, suggesting that serum TNF-*alpha* may rise in the early phase of the pneumonia, gastrointestinal obstruction, and other infectious/inflammatory complications [6]. Furthermore, we considered that endogenous TNF-*alpha* itself accounted for the majority of the elevated concentration of TNF-*alpha* at the time of pneumonia in this case. On the other hand, TNF-*alpha* concentrations were steady after the start of etanercept therapy until the cessation of the drug. This phenomenon might demonstrate that the TNF-*alpha* levels reflected the proper drug dosage in the course of etanercept treatment.

 In conclusion, although TNF-*alpha* and TNFR-II concentrations measured by ELISA might consist of the TNF-etanercept complex and even etanercept itself, these concentrations could reflect the microenvironments in the course of etanercept therapy. Monitoring TNF-*alpha* levels might provide much information in terms of avoiding serious adverse events and evaluating clinical efficacy.
